# “Off-label” Usage of an Oxidized Zirconium Femoral Head in Revision of a Total Hip Arthroplasty with Mechanically Assisted Crevice Corrosion and a Legacy Taper

**DOI:** 10.1016/j.artd.2021.01.012

**Published:** 2021-03-01

**Authors:** Andrew D. Lachance, Brian J. McGrory, Robert A. Christman

**Affiliations:** Tufts University School of Medicine, Boston, MA, USA; Division of Joint Replacements, Department of Surgery, Maine Medical Center, Portland, ME, USA; Department of Pathology, Maine Medical Center, Portland, ME, USA; Maine Joint Replacement Institute, Portland, ME, USA

**Keywords:** Revision, Total hip replacement (THR), Mechanically assisted crevice corrosion (MACC), Adverse local tissue reaction (ALTR), Oxidized zirconium

## Abstract

We present a case of a 72-year-old male with a history of a late 1980s metal-on-polyethylene total hip arthroplasty who presented with unilateral leg vascular compromise, joint pain, and stiffness and subsequently underwent revision for adverse local tissue reaction secondary to mechanically assisted crevice corrosion. His stable and extensively porous coated femoral implant had a legacy taper with no currently manufactured option for a non–Co-alloy femoral head. After shared decision-making with the patient, we opted to use an oxidized zirconium femoral head from another manufacturer with a similar taper during his revision surgery and documented that his vascular compromise resolved and his serum Co was undetectable 3 years after the revision.

## Introduction

Femoral head and stem trunnion modularity was introduced in the 1980s and is now widely accepted in total hip reconstruction. There was early recognition of tribocorrosion at this new head-neck interface soon after its introduction [1,2], but significant clinical failures have been more recently observed [3,4]. At the head bore-stem trunnion interface, synergistic fretting and corrosion mechanisms are known collectively as mechanically assisted crevice corrosion (MACC) [5,6]. Taper corrosion appears to be more common when dissimilar metals interface at the head-neck junction but has been reported with same metal combinations also [3,7,8]. The ionic, chemical, and particulate debris produced may result in a local inflammatory response described as adverse local tissue reaction (ALTR) [9,10]. This inflammatory process has for the most part been observed only in patients with a cobalt (Co) alloy femoral head [9].

Here, we present a case of MACC occurring in a patient with a metal-on-polyethylene modular hip prosthesis who presented with unilateral lower extremity swelling and large intrapelvic mass. Resolution was obtained with revision to an oxidized zirconium-on-polyethylene construct in what is, to our knowledge, the first description of the “off-label” usage of this particular product because a ceramic or non–Co-alloy head was not available for revision. A staged surgery to remove the intrapelvic mass was needed, but blood flow to the limb was restored and follow-up serum Co testing revealed undetectable levels, consistent with resolution of the patient’s MACC and lack of corrosion at the new head-neck junction.

## Case history

**Figure 1:**
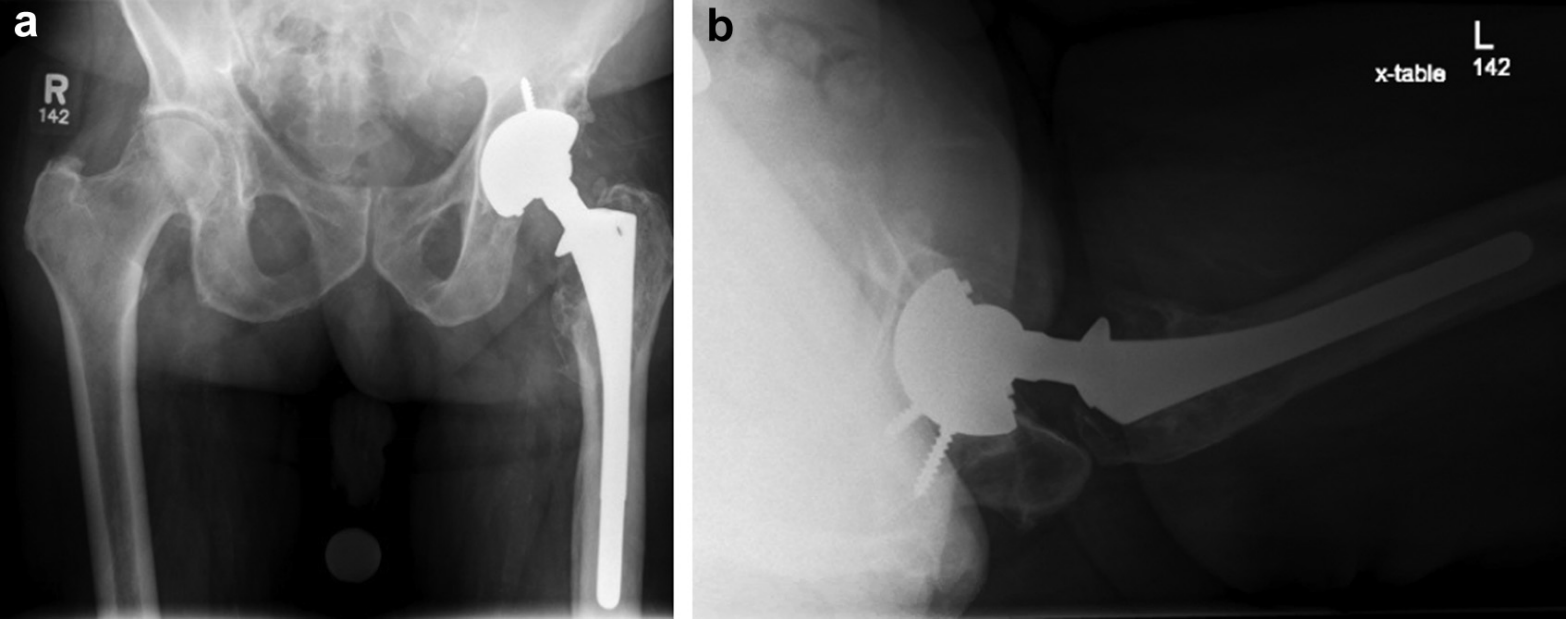
Anteroposterior (AP) pelvis (a) and lateral hip radiograph (b) showing left total hip replacement with extensively ingrown femoral stem, proximal femoral osteolysis, and well-fixed, noncemented acetabular component with minimal eccentricity.

**Figure 2:**
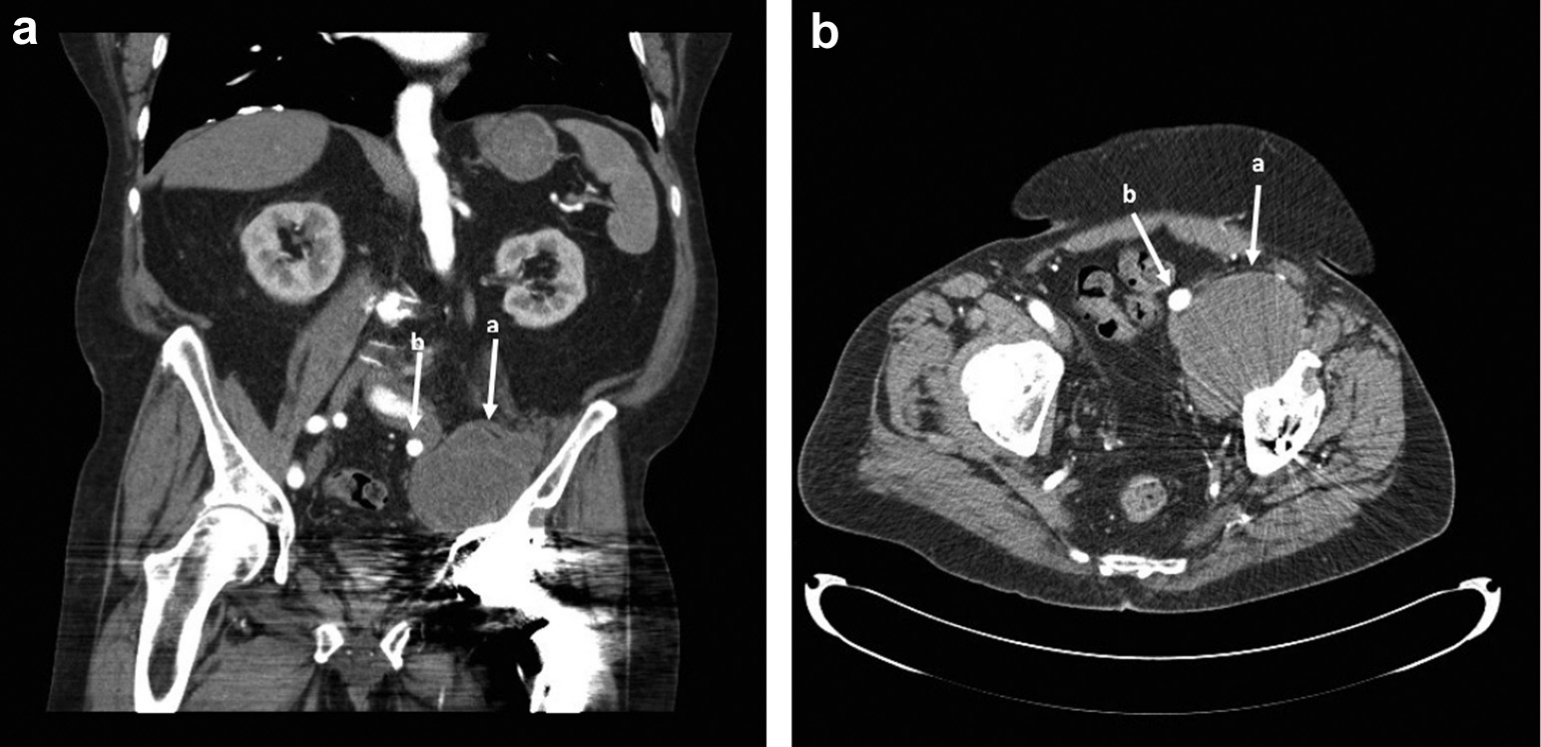
A contrast-enhanced angiographic computed tomography (CT) scan of the pelvis demarcating the left pelvis mass (a) in the iliac fossa that displaces the left common iliac artery (b) and vein medially. (a) Coronal view. (b) Axial view.

**Figure 3:**
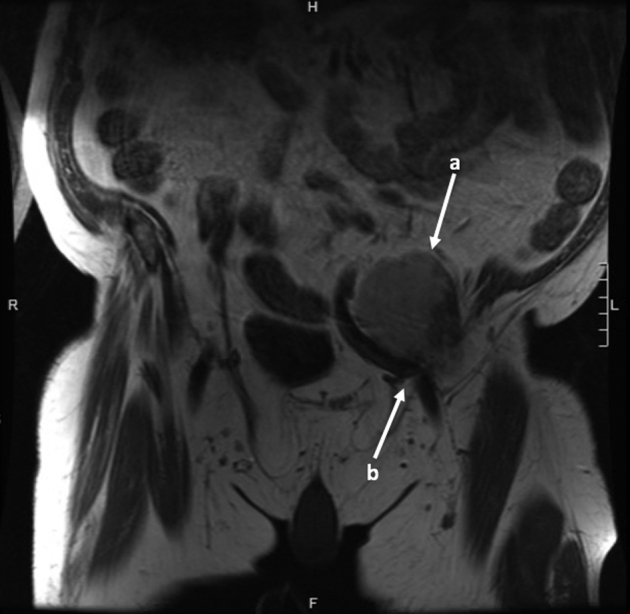
Metal artifact reduction sequence (MARS) magnetic resonance imaging (MRI) demonstrates a distinct anterior hip mass (a), emanating from the hip joint and encroaching on the anterior vascular structures (b).

The patient is a 72-year-old male presenting with left leg weakness and a pelvic mass 26 years after total hip replacement and 16 years after revision for polyethylene wear. He complained of 3 years of weakness and progressive neurovascular compromise and was evaluated by a board-certified vascular surgeon. He presented with symptoms of claudication pain and what was thought to be a pulsatile inguinal mass on examination; this was found to be a complex mass associated with his THA. His past medical history is significant for degenerative joint disease of the lumbar spine, hypertension, Parkinson's disease, prostate cancer with radiation therapy, scoliosis, squamous cell carcinoma in situ, and atrial fibrillation. The patient presented with neurovascular compromise of the left lower extremity, suspected to be due to a mass caused by ALTR originating from his hip arthroplasty. Radiographs (Fig. 1) showed a well-fixed, well-positioned noncemented THA, with some proximal femoral osteolysis thought to be related to his prior THA revision. A computed tomography scan of the pelvis delineated the left groin mass in the iliac fossa that displaced the left common iliac artery and vein (Fig. 2). Metal artifact reduction magnetic resonance imaging confirmed that the mass was related to the hip joint (Fig. 3). Vascular compromise was further supported by ultrasound showing an ankle-brachial index [11] of 0.50 on the affected side compared with 1.66 on the right.

Joint aspiration was negative for evidence of bacterial infection (no growth at 14 days; cell count not available because of cell debris), but serum Co and Cr levels were abnormally elevated. Manual cell count was inadvertently not ordered at the time of aspiration and was not thought to be so critical that the patient needed to undergo a second aspiration. Three months before revision, serum Co was 13 ppb (normal <0.3 ppb), and serum Cr was 5.3 (normal, 0.0-0.9 ppb). Complete blood count revealed a white blood cell count of 6900 (normal 4200-9900), and C-reactive protein was found to be 1.7 (normal, 0.0-8.0 mg/L). The patient was diagnosed with MACC of the trunnion-bore junction and secondary ALTR.

There did not appear to be significant eccentric polyethylene loss on radiographs (Fig. 1), although by medical records the polyethylene was not highly cross-linked and had been implanted 16 years prior. There was proximal femoral osteolysis, and this was thought to be related to polyethylene wear associated with his prior revision in 1998. From the patient’s prior surgeries, the implants were determined to be an anatomic medullary locking (AML) stem made from Co alloy with DePuy “large taper” Co alloy femoral head and a PFC Co alloy acetabulum shell with a high-molecular-weight polyethylene modular liner (DePuy Synthes, Warsaw, IN). Only cobalt-alloy femoral heads and 28-mm internal diameter polyethylene bearing surface components were available from the manufacturer for replacement at the time of revision.

After shared decision-making, the patient decided on total hip revision surgery urgently because of the symptoms of neurovascular compromise. Although we discussed a 2-team, 2-incision approach, we opted for revision arthroplasty first, with follow-up mass excision because of the patient’s comorbid conditions and the potential increased risks and complexities of the more prolonged single-stage surgery. A revision THA through a posterior approach was performed [cementing a cross-linked high molecular weight polyethylene (HMWPE)] in the well-fixed acetabulum [12,13] and impacting a non-cobalt 32-mm femoral head on the trunnion of the stem). The acetabulum polyethylene used was a Zimmer longevity melt-annealed cross-linked HMWPE (Zimmer Biomet, Warsaw, IN) cemented into the shell of the previous THA. Before cementing, the external surface of the liner was roughened with the use of a high-speed burr on the back table, and the internal cup surface was likewise roughened using a previously described gel technique to minimize particulate debris [14]. Mechanical roughening was performed to increase cement adhesion. Acetabular cementation was performed with one batch of DePuy bone cement with gentamicin (CMW; DePuySynthes, Warsaw, IN). The femoral component was revised to a 14/16 taper Oxinium (oxidized zirconium) +4 mm, 32-mm femoral head (Smith and Nephew, Inc., Memphis, TN), and impacted in line with the axis if the trunnion with 5 sharp blows of a 500-gram hammer hitting a straight plastic and metal head impactor [15]. These components were used in an off-label manner on both counts: the acetabulum using a previously described and acceptable technique to use a larger internal diameter cup and a cross-linked component [12,13]; and the femoral head in conjunction with the patient's 14/16 large taper DePuy stem with a previously undescribed technique to increase the available femoral head diameter and also use a non-cobalt implant because of his MACC. This was used to minimize corrosion and cobaltemia as the patient's Co level was already pathologically elevated. The polyethylene liner was demonstrated to be stable after cementation, and the femoral taper was found to engage after impaction.

The trunnion of the stem had mild discoloration at surgery (Goldberg grade 2, [16]), and this was cleaned and dried before implantation of the new head [17]. The head was not grossly loose, and there were no visible or palpable areas of loss of contour of the trunnion. The polyethylene liner of the cup was clearly worn and yellow, with flaking “white cracks” consistent with anisotropic oxidation [18], and this was removed with a previously described technique [19]. As this was a cobalt alloy shell, we therefore removed the central, so-called “manhole cover” as well as the 2 fixation screws that were slightly loose. No corrosion or obvious fretting was noted around the screw-shell junction.

A bacterial culture was obtained and ultimately negative. Osteolysis was noted in the proximal femur which was debrided intraoperatively. Frozen section pathology specimen was negative for acute inflammation. No definitive aseptic lymphocyte-dominant vasculitis-associated lesions (ALVALs) were noted, but this is not uncommon with MACC [10], as sample location and pathologist training are critical.

Postoperatively, the patient was started on low-dose warfarin for deep vein thrombosis prophylaxis. Three months later after his hip revision, his intrapelvic mass had not involuted. The offending mass was therefore excised by the vascular surgery team using a pelvic approach. Pathology showed no acute inflammation; however, there was abundant tissue necrosis with associated macrophages. Focal, perivascular collections of lymphocytes and plasma cells were also identified. Using Oxford criteria for ALVAL: necrosis 3+ (greater than 25%); inflammation (number of cells) 3+ (greater than 50); inflammation (tissue percent area) 1+ (less than 10 percent); lymphocyte cuffing focal (less than 5 cells thick). Oxford ALVAL score [20] was therefore 1+. Cultures from the mass demonstrated coagulase-negative, gram-positive cocci in broth only. Although this was considered likely a contaminant based on his history, examination, and cultures (as well as the concurrent pathology), it was treated with a 72-hour course of intravenous vancomycin until blood cultures were negative and the pathology reading was finalized [21]. Because MACC is thought to increase risk of infection postoperatively [22], and this might be considered a class 4 [23] wound by nature of necrosis associated with MACC [24], this approach was thought to be most advantageous. Eighteen months after this procedure, ankle-brachial index improved to 1.74 compared with 0.5 nine months preoperatively. Dorsalis pedis and posterior tibial pulses were noted to be significantly improved postoperatively.

**Figure 4:**
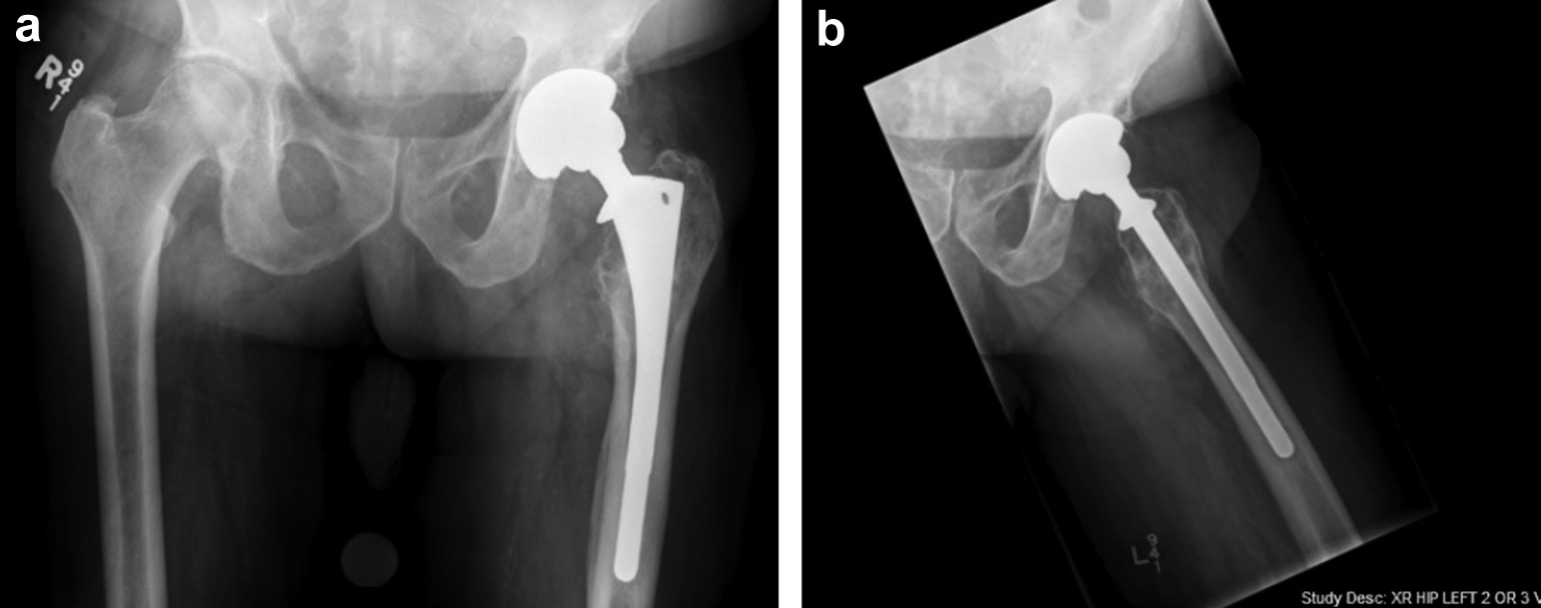
Antero-posterior (a) and frog lateral (b) 1-year follow-up radiographs, demonstrating a stable appearance and no new osteolysis or signs of loosening.

Radiographs (Fig. 4) and clinical examination at 1 year postoperatively were stable.

The patient did sustain 3 posterior hip dislocations postoperatively between 4 months and 14 months postoperatively. Each occurred with flexion and internal rotation of the hip and was treated with closed reduction, patient education, prolonged posterior hip precautions, and activity modification. The instability resolved, and the patient has not had any further dislocations since 14 months postoperatively until final clinical recheck at 5 years.

The cobalt levels rose when measured 3.5 months postoperatively from the hip revision to 21.8 ppb (Cr 6.9 ppb), and this was thought to be secondary to the preparation of the well-fixed Co-alloy acetabular shell. Three years postoperatively, however, serum Co was undetectable, and serum Cr was 1.2 ppb.

Five years after his revision, the patient is pain free and has no functional deficits related to his hip. His Parkinson’s disease has progressed, and he uses a cane for balance. He has no vascular symptoms.

The patient has consented to submitting this manuscript for publication.

## Discussion

ALTR in an older failed THA can be multifactorial, and all sources should be considered and addressed at the time of revision. Preoperative evaluation with serum Co and Cr is the best way to screen for possible MACC, and if the Co is elevated greater than 1 ppb [25,26], the taper should be evaluated and a Co-alloy revision head avoided at the time of surgery. It is important to note that severity of visible corrosion does not always correlate with serum Co [27] and that visual inspection alone is not perfect in determining corrosion [28]. Importantly, if a Co alloy head is used at revision, recurrence of symptomatic MACC is likely [3,29].

The AML stem, a bead-coated, Co-alloy, noncemented prosthesis, was introduced initially as a nonmodular implant in 1977 and became available for general use after U.S. Food and Drug Administration approval in 1983 [30,31]. Later, in 1985, femoral head modularity was introduced to aid in fine-tuning femoral offset, leg length, stability, and implant inventory issues. This component, with a relatively large, stiff, and short trunnion, has enjoyed large-scale clinical success in the long term [31,32]. A recent study of well-functioning implants from a small cadaver cohort did demonstrate mild corrosion and MACC in 29% and severe corrosion in 2% [33]. The damage score was correlated with length of implantation. Corrosion symptoms and need for revision are therefore rare but not unheard of for this implant.

For contemporary THA stems, a range of ceramic primary and revision heads with titanium (Ti) sleeves are available from many manufacturers. With both noncemented Ti and Co-alloy stems, this approach has been demonstrated to decrease Co production at the taper, and therefore cobaltemia and MACC [3,9,29]. Legacy trunnion sizes from older designs or implants no longer in production may offer a challenge to the revision surgeon to find an appropriate noncobalt alloy femoral head. While companies often support such revisions with Co-alloy heads, non–Co-alloy heads may be limited in size allotment and material [eg, DePuy Synthes, Inc. (Warsaw, IN) only offers alumina heads of limited neck lengths and head sizes for the Sivash-Range of Motion (SROM) stem.] or not available at all (eg, Zimmer Biomet, Inc. [Warsaw, IN] does not offer a 6-degree taper revision head in a non-Co material.) [34]. The DePuy Synthes, Inc. (Warsaw, IN) large taper, as is exemplified by this case, is yet another example of lack of non-Co head availability.

Surgeons could consider manufacturing a custom Ti sleeve for use with a large-bore prefabricated ceramic head [34] in such situations. However, this requires a complex process approved by the Food and Drug Administration for humane usage, may be quite expensive, and does not allow multiple options for intraoperative circumstances that may arise. In addition, for large tapers such as the 14/16 used in this case, prefabricated ceramic heads may not be available. Likewise, the use of a ceramic head without at Ti (Ti) sleeve is thought to be at high risk for fracture [35] and is “off-label,” although it has been performed successfully with minor existing trunnion damage and followed up to mid-term [36,37]. “Off-label” usage of a femoral head from another manufacturer is also a possibility but may be difficult because even tapers of the same nominal size may be different [38] and reliable seating is not guaranteed [39]. One variation of this approach is a system such as BioBall (Merete Medical, Berlin, GR) if the trunnion is a variation of a 12/14 taper (14/16 tapers are available in Europe only.). This company produces ceramic and metallic heads with a variety of sizes, offset bores, and lengths that can be used in an “off-label” manner with other manufacturer’s trunnions [40,41]. Although it would be ideal if revision femoral heads were from the same manufacturer as the stem (so the trunnion matches precisely), there may be rare cases when a surgeon may have to consider “off-label” usage with a femoral head from another manufacturer.

In our case, we used a 14/16 oxidized zirconium (Oxinium; Smith and Nephew, Memphis, TN) head for a corroded large (14/16) Depuy taper on a Co alloy well-fixed stem. Oxidized zirconium is Co free, compatible with Co alloy stems, does not fracture, and is corrosion-resistant [42]. In this case, it allowed a stable construct without removing the frail patient’s extensively coated stem, and corrosion resistance was documented with undetectable serum Co at 3.4 years. Also of note is the fact that the reversal of serum Co also confirms an acceptable taper-lock, as one would expect a poorly matched bore-trunnion combination to cause trunnion fretting, and Co from this source.

One interesting finding in our patient was a high serum Co detected at 3.5 months after THA revision. We hypothesize that this related to the preparation of the well-fixed acetabular shell for cementing. We used a high-speed burr to roughen the surface and likely caused Co debris despite our ultrasound-gel particle entrapment technique [14]. We demonstrate that this elevation completely resolved as the one-time bolus of Co alloy debris was excreted over time through the urine.

We present this case and acknowledge that the strategy highlighted may be considered to be necessary in very rare and infrequent circumstances. We think that in an urgent clinical situation when Food and Drug Administration compassionate usage for a custom implant is not practical based on timing and/or cost, and no other options are available, “off-label” usage of this particular 14/16 oxidized zirconium femoral head (32, +4) may be considered for usage with an AML large taper trunnion based on our short-term experience. We also note that continued surveillance of our patient is appropriate and ongoing and longer term follow-up will be important.

## Summary

MACC with a Co-alloy femoral head produces ionic, chemical and particulate debris that may cause ALTR. Appropriate treatment of this problem includes revision surgery to replace the femoral head with a non–Co alloy component, with debridement of the adverse local tissue. In most cases, a non–Co alloy revision femoral head is available from the femoral component manufacturer. In rare cases of a legacy trunnion, where such a head is not available, unique options may be called upon. One such option, based on the presented case history, is using a 14/16 Smith and Nephew Oxinium (oxidized zirconium) femoral head with a large taper on a DePuy AML stem. We show that at 5 years, the clinical status of a patient after such a revision is stable, and at 3 years, postrevision serum cobalt decreases from 13 ppb to undetectable levels.

## Conflict of interests

Dr. McGrory is a paid consultant for Smith and Nephew, Inc., the company that manufactures the Oxinium (oxidized zirconium) femoral head.

For full disclosure statements refer to https://doi.org/10.1016/j.artd.2021.01.012.

## Appendix A Supplementary data

Conflict of Interest Statement for Christman

Conflict of Interest Statement for Lachance

Conflict of Interest Statement for McGrory

## References

[1] Collier J.P., Surprenant V.A., Jensen R.E., Mayor M.B. (1991). Corrosion at the interface of cobalt-alloy heads on titanium -alloy stems. Clin Orthop Rel Res.

[2] Collier J.P., Mayor M.B., Williams J.M., Surprenant V.A., Surprenant H.P., Currier B.H. (1995). The tradeoffs associated with modular hip prostheses. Clin Orthop Rel Res.

[3] Cooper H.J., Della Valle C.J., Berger R.A. (2012). Corrosion at the head-neck taper as a cause for adverse local tissue reactions after total hip arthroplasty. J Bone Joint Surg.

[4] Hussey D., McGrory B.J. (2017). Ten-year cross-sectional study of mechanically assisted crevice corrosion in 1352 consecutive patients with metal-on-polyethylene total hip arthroplasty. J Arthroplasty.

[5] Gilbert J.L., Buckley C.A., Jacobs J.J. (1993). In vivo corrosion of modular hip prosthesis components in mixed and similar metal combinations. The effect of crevice, stress, motion, and alloy coupling. J Biomed Mater Res.

[6] Morlock M.M. (2015). The taper disaster-how could it happen?. Hip Int.

[7] Jennings J.M., Dennis D.A., Yang C.C. (2016). Corrosion of the head-neck junction after total hip arthroplasty. J Am Acad Orthop Surg.

[8] Weiser M.C., Lavernia C.J. (2017). Current concepts review: trunnionosis in total hip arthroplasty. J Bone Joint Surg.

[9] Jacobs J.J., Cooper H.J., Urban R.M., Wixson R.L., Della Valle C.J. (2014). What we know about taper corrosion in total hip arthroplasty. J Arthroplasty.

[10] McGrory B.J., Jacobs J.J., Kwon Y.-M., Fillingham Y.A. (2020). Standardizing terms for tribocorrosion-associated adverse local tissue reaction in total hip arthroplasty. Arthroplasty Today.

[11] Chongthawonsatid S., Dutsadeevettakul S. (2017). Validity and reliability of the ankle-brachial index by oscillometric blood pressure and automated ankle-brachial index. J Res Med Sci.

[12] Beaule P.E., Ebramzadeh E., LeDuff M., Prasad R., Amstutz H.C. (2004). Cementing a liner into a stable cementless acetabular shell: the double-socket technique. J Bone Joint Surg.

[13] Bedard N., Tetreault M., Hanssen A., Lewallen D.G., Trousdale R.T. (2020). Intermediate to long-term follow-up of cementing liners into well-fixed acetabular components. J Bone Joint Surg.

[14] McGrory A.C., Replogle L., Endrizzi D.E. (2016). Ultrasound gel minimizes third body debris with partial hardware removal in joint arthroplasty. Arthroplasty Today.

[15] McGrory B.J., Ng E. (2018). No consensus for femoral head impaction technique in surgeon education materials from orthopaedic implant manufacturers. J Arthroplasty.

[16] Goldberg J.R., Gilbert J.L. (2003). In vitro corrosion testing of modular hip tapers. J Biomed Mater Res B Appl Biomater.

[17] McGrory B.J., McKenney B.R. (2016). Revision for taper corrosion at the head-neck junction: pearls and pitfalls. Curr Rev Musculoskel Med.

[18] Muratoglu O.K., Mounib L., McGrory B.J., Bragdon C.R., Jasty M., Harris W.H. (1998). Anisotropic oxidation and radial cracksin retrieved acetabular components. 44th annual Orthopaedic Research Society. New Orleans, Louisianna, USA.

[19] Villanueva-Martinez M., Rios-Luna A., Pereiro-De Lamo J., Fahandez-Saddi H., Benito-Del Carmen F. (2007). Techical Note: a simple polyethylene liner extraction method in hip arthroplasty revision surgery. Eur J Orthop Surg Traumatol.

[20] Grammatopoulos G., Pandit H., Kamali A. (2013). The correlation of wear with histological features after failed hip resurfacing arthroplasty. J Bone Joint Surg.

[21] Smith E.B., Cai J., Wynne R., Maltenfort M., Good R.P. (2014). Performance characteristics of Broth-only cultures after revision total joint arthroplasty. Clin Orthop Rel Res.

[22] McGrory B.J., Jorgensen A. (2017). High early major complication rate after revision for mechanically associated crevice corrosion in metal-on-polyethylene total hip arthroplasty. J Arthroplasty.

[23] Herman T., Bordoni B. (2020). Wound classification. Last update 05/14/2020.

[24] Eltit F., Assiri A., Garbuz D. (2017). Adverse reactions to metal on polyethylene inplants: highly destructive lesions related to elevated concentrations of cobalt and chromium in synovial fluid. J Biomed Mater Res A.

[25] Fillingham Y.A., Della Valle C.J., Bohl D.D. (2017). Serum metal levels for the diagnosis of adverse local tissue reaction secondary to corrosion in metal-on-polyethylene bearing total hip arthroplasty. J Arthroplasty.

[26] Kwon Y.M., MacAuliffe J., Arauz P.G., Peng Y. (2018). Sensitivity and specificity of metal ion level in predicting adverse local tissue reactions due to head-neck taper corrosiuon in primary metal-on-polyethylene total hip arthroplasty. J Arthroplasty.

[27] McGrory B.J., MacKenzie J., Babikian G. (2015). A high prevalence of corrosion at the head-neck taper with contemporary Zimmer non-cemented hip components. J Arthroplasty.

[28] Hothi H.S., Kendoff D., Lausmann C. (2017). Clinically insignificant trunnionosis in larger-diameter metal-on-polyethylene total hip arthroplasty. Bone Joint Res.

[29] Cooper H.J., Urban R.M., Wixson R.L., Meneghini R.M., Jacobs J.J. (2013). Adverse local tissue reaction arising from corrosion at the femoral neck-body junction in a dual-taper stem with a cobalt-chromium modular neck. J Bone Joint Surg.

[30] Engh C. (2020). Extensively coated stems. Musculoskeletal Key.

[31] Riviere C., Grappiolo G., Engh C.A. (2018). Long-term bone remodelling around "legendary" cementless femoral stems. EFORT Open Rev.

[32] Engh C.A., Culpepper W.J. (1997). Femoral fixation in primary total hip arthroplasty. Orthopaedics.

[33] Lange J., Wach A., Koch C.N. (2018). Do well-functioning THAs retrieved at autopsy exhibit evidence of fretting and corrosion. Clin Orthop Rel Res.

[34] Leibiger T., McGrory B.J. (2015). Custom titanium sleeve for surgical treatment of mechanically assisted crevice corrosion in the well-fixed, non-contemporary stem in total hip arthroplasty. Arthroplasty Today.

[35] Vogel D., Hembus J., Jackzis M., Bolte V., Bader R. (2020). Influence of different damage patterns of the stem taper on fixation and fracture strength of ceramic ball heads for total hip replacement. Biomed Res Int.

[36] Hannouche D., Delambre J., Zadegan F., Sedel L., Nizard R. (2010). Is there a risk in placing a ceramic head on a previously implannted trunion?. Clin Orthop Rel Res.

[37] Kim Y., Park J., Kim J. (2018). Adapter sleeves are not needed to reduce the risk of fracture of a new ceramic head implanted on a well-fixed stem. Orthopedics.

[38] Berstock J., Whitehouse M., Duncan C. (2018). Trunnion corrosion: what surgeons need to know in 2018. J Bone Joint Surg.

[39] Mueller U., Panzram B., Braun S., Sontag R., Kretzer J.P. (2017). Mixing of head-stem components in total hip arthroplasty. J Arthroplasty.

[40] Kock H., Cho C., Buhl K., Hillmeier J., Huber F. (2020). Long-term outcome after revision hip arthroplasty with the BioBall adapter system in multimorbid patients. J Orthop Translation.

[41] Novoa C., Citak M., Zahar A., Lopez R., Gehrke T., Rodrigo J. (2018). The Merete BioBall system in hip revision surgery: a systematic review. Orthop Traumatol Surg Res.

[42] Cartner J., Aldinger P., Li C., Collins D. (2017). Characterization of femoral head taper corrosion features using a 22-year retrieval database. HSS J.

