# Supplementary material for: “Off-label” Usage of an Oxidized Zirconium Femoral Head in Revision of a Total Hip Arthroplasty with Mechanically Assisted Crevice Corrosion and a Legacy Taper
Source: Arthroplast Today. 2021 Mar 1;8:69–73. doi: 10.1016/j.artd.2021.01.012 (PMC7930501; doi:10.1016/j.artd.2021.01.012)
Supplement: Conflict of Interest Statement for Lachance [file mmc2.pdf]

CONFLICT OF INTEREST STATEMENT

American Association of Hip and Knee Surgeons

(Adopted from the American Academy of Orthopaedic Surgeons disclosure statement)

The following form **must be filled out completely and submitted by each author (example, 6 authors, 6 forms). All items require a response. If there is no relevant disclosure for a given item, enter "*None.*"**

Manuscript Title: Off-label" usage of an oxidized zirconium femoral head in revision of a total hip arthroplasty with mechanically assisted crevice corrosion and a legacy taper

1. Royalties from a company or supplier (The following conflicts were disclosed)

None

2. Speakers bureau/paid presentations for a company or supplier (The following conflicts were disclosed)
None

3A. Paid employee for a company or supplier (The following conflicts were disclosed)
None

3B. Paid consultant for a company or supplier (The following conflicts were disclosed)
None

3C. Unpaid consultants for a company or supplier (The following conflicts were disclosed)
None

4. Stock or stock options in a company or supplier (The following conflicts were disclosed)
None

5. Research support from a company or supplier as a Principal Investigator (The following conflicts were disclosed)
None

6. Other financial or material support from a company or supplier (The following conflicts were disclosed)
None

7. Royalties, financial or material support from publishers (The following conflicts were disclosed)
None

8. Medical/Orthopaedic publications editorial/governing board (The following conflicts were disclosed)
None

9. Board member/committee appointments for a society (The following conflicts were disclosed)
None

Each author must sign AND print or type his/her name, date and submit a separate form

In addition, one BLINDED Conflict of Interest form (no author names used) should be submitted per manuscript with all author disclosures.

Andrew D. Lachance	Andrew D. Lachance	9/19/20
Author Name (Print or Type)	Author Signature	Date
